# Korea’s 2024 reduction in medical research output amid physician residents’ resignation

**DOI:** 10.12771/emj.2025.00381

**Published:** 2025-04-17

**Authors:** Jeong-Ju Yoo, Hyun Bin Choi, Young-Seok Kim, Sang Gyune Kim

**Affiliations:** 1 Division of Gastroenterology and Hepatology, Department of Internal Medicine, Soonchunhyang University Bucheon Hospital, Bucheon, Korea; 2 Department of Internal Medicine, Soonchunhyang University Bucheon Hospital, Bucheon, Korea

In February 2024, the Korean government announced it would increase medical school admissions by 2,000 seats—a 65.4% rise from the existing quota of 3,058 [1]. This policy, introduced with minimal consultation with the medical community and lacking a clear scientific rationale, prompted widespread protests [2]. More than 90% of resident physicians resigned, plunging the healthcare system into disarray [3-5]. Over a year later, the dispute between the government and the medical profession remains unresolved, threatening Korea’s healthcare infrastructure and academic research productivity.

In Korea, resident physicians constitute nearly 40% of the physician workforce in university hospitals. They bear primary responsibility for inpatient care—including prescriptions, diagnostic tests, and procedures—and play a crucial role in patient monitoring and daily round preparations. Moreover, they assist specialists in the emergency department, intensive care units, and operating rooms. This high reliance on residents meant that their absence overwhelmed university hospitals, forcing faculty members to assume the full spectrum of clinical duties. Professors, who previously balanced patient care, education, and research, have shifted almost entirely to clinical responsibilities, resulting in a marked decline in academic output. Concurrent reductions in government‐funded research grants during this period likely exacerbated the downturn in scholarly productivity [6]. The combined impacts of funding cuts and workforce shortages have placed unprecedented strain on university‐based research systems.

A downward trend in medical research publications was already evident before 2024. Between 2022 and 2023, publication counts fell modestly by 2.60% in the Web of Science database (20,247 vs. 20,788) and by 5.56% in the Embase database (18,977 vs. 20,094). These databases were chosen for their comprehensive coverage of peer‐reviewed medical journals.

The ongoing conflict appears to have significantly accelerated this decline, with a further reduction of 12.01% (17,816 vs. 20,247) in Web of Science and 12.50% (16,604 vs. 18,977) in Embase in 2024 compared with 2023. In contrast, a similar protest in 2020, when resident physicians opposed the expansion of medical school admissions, lasted only one month (August–September) and had minimal impact on research output (Fig. 1, Supplement 1). This sharp decline in medical research contrasts with trends in other fields: based on Web of Science data, medical publications fell by 12.01% in 2024 versus 2023, while publications in the natural sciences and engineering rose by 3.14% and 5.05%, respectively (Fig. 2, Supplement 2). The specific subject categories for natural sciences and engineering used in this comparison are detailed in Supplement 3.

The decline in research output varied widely by specialty. Hematology (–54.2%; 175 vs. 382), rheumatology (–42.7%; 160 vs. 279), dermatology (–39.3%; 300 vs. 494), respiratory medicine (–30.4%; 562 vs. 807), and pediatrics (–29.4%; 266 vs. 377) experienced the steepest drops (Fig. 3, Supplement 4). These fields depend heavily on resident physicians for both clinical service and academic activities. In contrast, some specialties saw modest increases: transplantation (+3.7%; 280 vs. 270), pathology (+4.7%; 179 vs. 171), urology & nephrology (+8.3%; 471 vs. 435), ophthalmology (+9.4%; 430 vs. 393), and critical care medicine (+21.3%; 182 vs. 150). These areas may have been buffered by lower reliance on residents and more established physician assistant systems.

The broader implications extend beyond research productivity. The disruption of clinical workflows has compromised patient care, particularly in tertiary hospitals, where resident physicians play a critical role. Furthermore, the government’s response has exacerbated tensions. On December 3, 2025, the Korean President declared martial law, mandating that resident physicians return to their duties within 48 hours—a measure widely criticized as an authoritarian overreach [7]. The decree stated:

“All medical professionals, including medical residents (resident physicians) who are currently striking or have left their medical posts, must return to their duties within 48 hours and perform their roles diligently. Failure to comply will result in punishment under martial law.”

The inflammatory rhetoric has further deepened the rift between the government and the medical community, destabilizing the healthcare system. It underscores the indispensable role of resident physicians in sustaining Korea’s clinical and academic infrastructures. The unilateral enactment of a policy with such far‑reaching consequences—without stakeholder engagement—has inflicted serious harm on both patient care and medical research. An immediate resolution is critical to restore stability and avert lasting damage to Korea’s global reputation for healthcare excellence and research innovation. Even if residents return, academic productivity is unlikely to rebound quickly: the prolonged disruption has already caused substantial delays in ongoing studies, weakened mentoring frameworks, and significantly increased faculty clinical workloads—factors that collectively foreshadow a sustained downturn in medical research output.

Future efforts should prioritize collaborative policymaking to rebuild trust between the government and the medical profession. This may include mediated dialogues with medical associations, incentives for resident‐physician retention, and the restoration of research funding to offset long‑term academic losses. Further studies are warranted to evaluate the lasting impact of these conflicts on patient outcomes and healthcare delivery.

This study has several limitations. In Fig. 3 (Supplement 4), we analyzed the number of published articles by specialty using subject categories from the Web of Science database. However, individual journals can be indexed under multiple categories in Web of Science; in such cases, we included each journal in all assigned categories rather than designating a single primary classification. Similarly, in Fig. 2, we grouped subject categories under the broader fields of natural sciences and engineering for comparative analysis. Nevertheless, some categories legitimately span both disciplines and were thus included in both. This methodological approach may have resulted in partial duplication of counts across disciplines, which should be considered when interpreting the results.

Korea’s healthcare system, long regarded as a model of efficiency and excellence, now stands at a critical crossroads. Resolving this crisis requires not only addressing immediate challenges but also fostering a culture of collaboration and mutual respect to safeguard the future of healthcare and medical research in the country.

## Figures and Tables

**Fig. 1.**
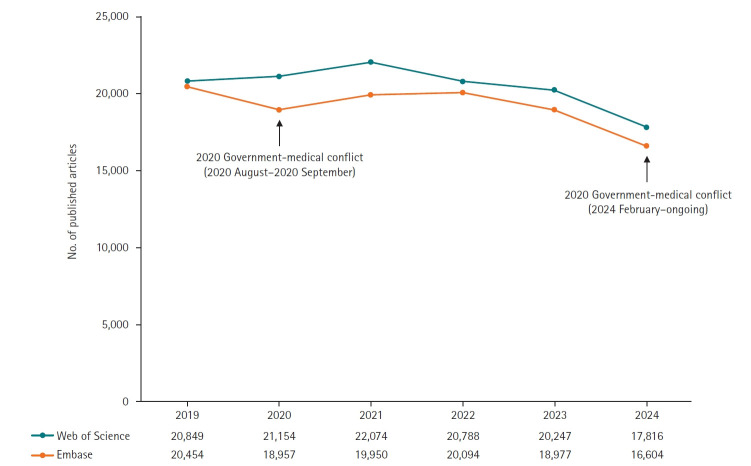
Number of published medical articles by authors with affiliations in Korea (2019–2024) in Web of Science and Embase.

**Fig. 2.**
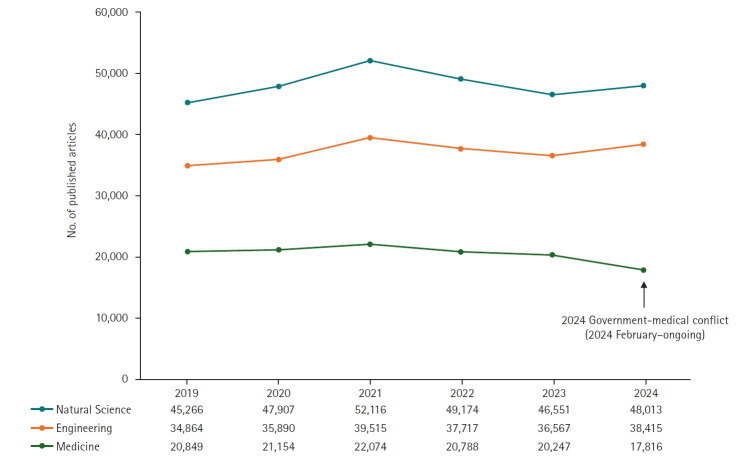
Number of published articles by authors with affiliations in Korea in natural sciences, engineering, and medicine (2019–2024) in Web of Science.

**Fig. 3.**
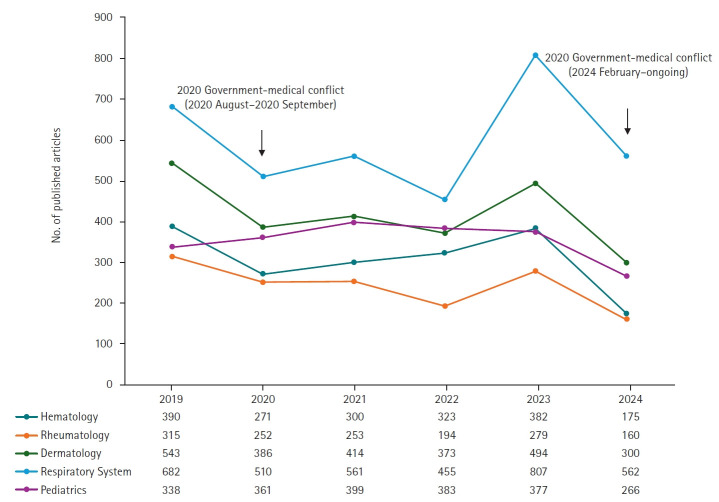
Number of published articles by authors with affiliations in Korea (2019–2024). Top 5 specialties with the highest reduction rates in 2024 compared to 2023.

## Supplementary materials

Supplementary files are available from Harvard Dataverse: https://doi.org/10.7910/DVN/P3PWGY

Supplement 1. Number of published articles by authors with affiliations in Korea (2019–2024) in Web of Science and Embase.

Supplement 2. Number of published articles by authors with affiliations in Korea in Natural Sciences, Engineering, and Medicine (2019–2024) based on Web of Science.

Supplement 3. The categories of natural sciences and engineering used for this comparative analysis.

Supplement 4. Number of published articles by authors with affiliations in Korea across specialties (2019–2024) based on Web of Science.
